# Indocyanine Green-Loaded Liposomes-Assisted Photoacoustic Computed Tomography for Evaluating In Vivo Tumor Penetration of Liposomal Nanocarriers

**DOI:** 10.3390/mi15010090

**Published:** 2023-12-30

**Authors:** Chenjun Wu, Qi Sun, Xiangdong Liu, Xin Sun, Zeyu Chen, Han Shan

**Affiliations:** 1 State Key Laboratory of Precision Manufacturing for Extreme Service Performance, College of Mechanical and Electrical Engineering, Central South University, Changsha 410083, China; 2 Changjun Riverside Middle School, Changsha 410023, China; 3 Department of Biomedical Engineering, University of Southern California, Los Angeles, CA 90089, USA

**Keywords:** indocyanine green, liposomes, tumor penetration, photoacoustic imaging, microfluidic mixing

## Abstract

Liposomes possess the potential to enhance drug solubility, prolong the duration of circulation, and augment drug accumulation at the tumor site through passive and active targeting strategies. However, there is a lack of studies examining the in vivo tumor penetration capabilities of liposomes of varying sizes, which hampers the development of drug delivery systems utilizing liposomal nanocarriers. Here, we present an indocyanine green (ICG)-loaded liposomes-assisted photoacoustic computed tomography (PACT) for directly evaluating the tumor penetration ability of liposomal nanocarriers in vivo. Through the utilization of microfluidic mixing combined with extrusion techniques, we successfully prepare liposomes encapsulating ICG in both large (192.6 ± 8.0 nm) and small (61.9 ± 0.6 nm) sizes. Subsequently, we designed a dual-wavelength PACT system to directly monitor the in vivo tumor penetration of large- and small-size ICG-encapsulated liposomes. In vivo PACT experiments indicate that ICG-loaded liposomes of smaller size exhibit enhanced penetration capability within tumor tissues. Our work presents a valuable approach to directly assess the penetration ability of liposomal nanocarriers in vivo, thereby facilitating the advancement of drug delivery systems with enhanced tumor penetration and therapeutic efficacy.

## 1. Introduction

**Scheme 1:**
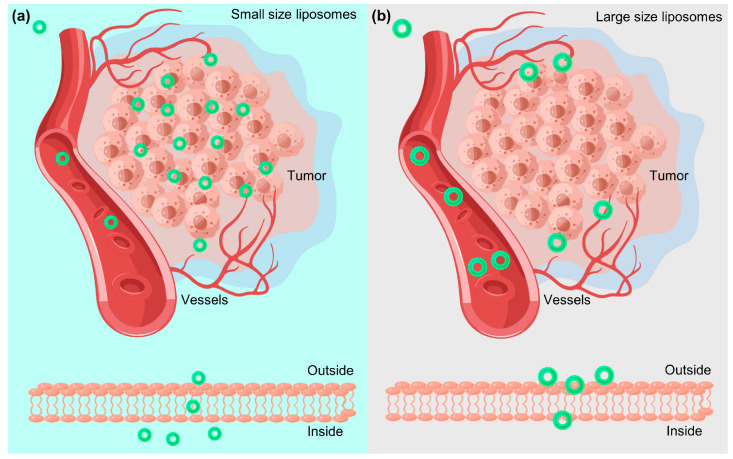
Schematics showing a comparison of the tumor penetration performance of (**a**) small-size liposomes and (**b**) large-size liposomes. The figure is produced by Figdraw 2.0 software.

The development of efficient drug delivery systems is crucial for enhancing the therapeutic efficacy of anticancer agents while minimizing their adverse effects on healthy tissues [1,2,3,4]. Among various drug delivery platforms, liposomes have gained significant attention due to their unique properties, such as biocompatibility, tunable size, and the ability to encapsulate both hydrophilic and hydrophobic drugs [5,6,7]. Liposomes can improve drug solubility, prolong circulation time, and enhance drug accumulation at the tumor site through passive and active targeting strategies [8,9]. Passive targeting relies on the enhanced permeability and retention (EPR) effect, which is a phenomenon associated with the leaky vasculature and poor lymphatic drainage in solid tumors [10]. This effect allows liposomes to preferentially accumulate in tumor tissues, offering a promising strategy for drug delivery. Of note, the size of liposomes plays an important role in tumor penetration [11,12]. As shown in Scheme 1, it has previously been envisioned that liposomes with smaller size have a higher delivery efficiency and greater toxicity for cancer cells. However, there is a lack of studies investigating the tumor penetration of liposomes with different sizes in vivo, limiting the development of drug delivery systems based on liposomal nanocarriers. Hence, there is a need to develop an innovative approach to directly assess the penetration ability of liposomal nanocarriers within the tumor sites.

Fluorescence imaging can effectively monitor the accumulation of drugs in tumors [13,14]. However, this traditional method cannot obtain the structural information in deep tissues, and it is difficult to effectively image the drug distribution in the internal structure. Photoacoustic imaging (PA) is an emerging imaging modality that integrates the advantageous features of ultrasound imaging, including high spatial resolution, with the sensitivity and specificity of optical imaging. Consequently, PA has emerged as a valuable tool for visualizing and characterizing drug distribution in deep tissues [15,16,17,18]. Within the realm of PA-based techniques, photoacoustic computed tomography (PACT) exhibits significant potential for clinical applications, such as whole-brain imaging [19], functional imaging of tumors [20], and therapy evaluation [21]. In comparison to alternative imaging modalities such as magnetic resonance imaging and X-ray computed tomography, PACT offers the advantages of being radiation-free and relatively cost-effective. In recent times, there has been a notable surge in the utilization of contrast agents in PACT imaging, owing to their ability to enhance signal-to-noise ratios and signal amplitudes [22]. Nonetheless, the preparation process for liposomal contrast agents is relatively intricate, thereby impeding the efficiency of PACT detection.

Microfluidic mixing technology has emerged as a powerful tool for the controlled synthesis of liposomal nanocarriers [23,24,25]. Compared to bulk methods, the microfluidic synthesis method exhibits uniform size distribution and good repeatability between batches [18,24]. During the mixing process, a stream of ethanol-containing lipids is mixed with a flow of aqueous buffer with a fixed flow rate ratio (FRR). Then, the dilution of ethanol induces the lipids to form self-assembled liposomes rapidly [26,27]. Therefore, liposomal PA contrast agents can be efficiently prepared by using microfluidic mixing, contributing to both greater sensitivity and greater accuracy in the detection of PACT.

In this study, we present an indocyanine green (ICG)-encapsulated liposomes-assisted PACT system to in vivo monitor the tumor penetration of drug-loaded liposomes with different sizes. ICG, as a drug approved by the U.S. Food and Drug Administration (FDA), was selected as a contrast agent due to its excellent imaging capabilities [28]. Specifically, ICG has peak absorption and emission wavelengths in the near-infrared region, typically around 780–805 nm and 805–820 nm, respectively. This is advantageous because NIR light can penetrate biological tissues more deeply than shorter-wavelength light, allowing for better imaging depth. Free ICG presents challenges in in vivo PA imaging due to its limited blood residence time and inadequate penetration of biological barriers. The utilization of ICG-loaded nanoparticles has garnered attention in biomedical imaging applications. Although a variety of nanoparticles (e.g., PLGA, SiO_2_, ZIF-8) have been developed to load ICG [29,30,31], poor biocompatibility hinders their clinical applications. By using microfluidic mixing combined with extrusion, we successfully prepared large- or small-size ICG-encapsulated liposomes. In vivo PACT experiments demonstrated that small-size ICG-loaded liposomes had a stronger penetration ability in tumor tissues. Our work provides an efficient and reliable strategy to directly evaluate the tumor penetration of liposomal nanocarriers in vivo and can contribute to the development of more effective drug delivery systems with improved tumor penetration and therapeutic efficacy.

## 2. Materials and Methods

### 2.1. Materials

1,2-Dipalmitoyl-sn-glycero-3-phosphocholine (DPPC), 1,2-distearoyl-sn-glycero-3-phosphoethanolamine-N-[amino(polyethylene glycol)2000] (DSPE-PEG2000), and cholesterol were obtained from Shanghai AVT Pharmaceutical Technology Co., Ltd., Shanghai, China. ICG was purchased from Shanghai Bidepharm Technology Co., Ltd., Shanghai, China. Ethanol was purchased from Hunan Huihong Reagent Co., Ltd., Changsha, China. Phosphate-buffered saline (PBS) (0.01 M, pH 7.4) was obtained from Codow Chemical Co., Ltd., Guangzhou, China. Polycarbonate (PC) membranes with 100 and 50 nm pores were obtained from Avanti Polar Lipids Inc., Alabaster, AL, USA. Dialysis bags were obtained from Hunan Yibo Biological Co., Ltd., Changsha, China. The yellow rigid resin was purchased from BMF Precision Technology Inc., Shenzhen, China. Microfluidic tapes were a gift from 3M China Co., Ltd., Shanghai, China. The 200-mesh copper grids were obtained from Zhengzhou Yasheng Electronic Technology Co., Ltd., Zhengzhou, China, and 2% phosphotungstic acid negative staining solution was obtained from Beijing Solarbio Science & Technology Co., Ltd., Beijing, China.

### 2.2. Microfluidic Mixer Fabrication

Our microfluidic chip was produced using an advanced high-resolution projection micro stereolithography (PμSL) 3D printer (microArch™ S140, BMF Precision Technology Inc., Shenzhen, China). Initially, the features of the microfluidic device were meticulously designed using Solidworks 2020. Subsequently, the 3D model was imported into a slicer software to generate precise cross-sectional images. The microfluidic device was then printed layer by layer by utilizing UV light at a 405 nm wavelength and employing the cross-sectional images as a dynamic mask. To better observe the flow profile, we selected a yellow, rigid, and transparent resin as our preferred material for device fabrication. After the printing process, the 3D-printed device was immersed in ethanol for a duration of 5 min to eliminate any uncured resin. Subsequently, it underwent a 3 min treatment with UV light to enhance its overall strength and rigidity. Our microfluidic mixing chip possessed dimensions of 17 × 10 × 2 mm^3^ and contained a mixing microchannel with a width of 0.2 mm and a depth of 0.15 mm. The inlet and outlet had a diameter of 1.6 mm and a depth of 2 mm. The 3D-printed microfluidic chip was sealed with a pressure-sensitive adhesive tape (Microfluidic tape, 3M). Finally, polytetrafluoroethylene (PTFE) tubes were inserted into the inlet and outlet and sealed with UV-curing glue.

### 2.3. Preparation of Large- and Small-Size ICG-Loaded Liposome

Large-size ICG-loaded liposome (L-ICG-Lipo) was directly prepared by our microfluidic mixing chip. In the formulation of liposomes, the selection of specific lipids plays a crucial role in determining the properties and functions of the liposomal system. We thoughtfully selected the formulation of DPPC, DSPE-PEG2000, and cholesterol for liposome preparation. DPPC is a phospholipid that contributes to the structural integrity and stability of the liposomal bilayer. DSPE-PEG2000 imparts stealth properties to liposomes, reducing their recognition and clearance by the immune system. Cholesterol is an essential component that modulates the fluidity and stability of the liposomal membrane. Briefly, we dissolved DPPC, DSPE-PEG2000, and cholesterol in ethanol at a molar ratio of 80/5/15. Following that, ICG was dissolved in PBS (0.01 M, pH 7.4) at a concentration of 0.1 mg/mL. Afterward, dual-channel syringe pumps (LD-P2020II, Shanghai LANDE Medical Equipment Co., Ltd., Shanghai, China) were used to introduce lipid solution (20 mM) and PBS to the microfluidic mixer with a buffer:ethanol FRR of 4 and a constant total flow rate (TFR) of 10 mL/h. To prepare small-size ICG-loaded liposome (S-ICG-Lipo), ICG-loaded liposomes were prepared as above, and the L-ICG-Lipo were passed through 0.1 and 0.05 μm PC membrane filters 11 times (Avanti Polar Lipids, Alabaster, AL, USA) in a liposome extruder (Liposofast, AVESTIN, Inc., Ottawa, ON, Canada). The prepared L-ICG-Lipo and S-ICG-Lipo were dialyzed against PBS for 24 h in a dialysis bag with a molecular mass cut of 14,000 Da to remove the excess organic solvents and free ICG molecules. The dialysis media were changed with fresh PBS at least three times. The ICG concentration in L-ICG-Lipo and S-ICG-Lipo was quantified using a UV–vis spectrophotometer (N4S, INESA Analytical Instrument Co., Ltd., Shanghai, China). The absorbance of a series of free ICG in ethanol was measured, and the standard calibration plot was obtained by selecting the absorbance maximum (Figure S1). Ethanol, a widely used solvent for liposome disruption, was used to dilute L-ICG-Lipo and S-ICG-Lipo at a ratio of 15:1. The encapsulation efficiency (EE) of ICG-loaded liposomes in ethanol was determined by measuring their absorbance and comparing it to a standard calibration plot. The EE was calculated using the equation:

EE = (E_drug_/T_drug_) × 100% (1)

where E_drug_ represents the liposome-associated amount of drug, and T_drug_ represents the total amount of drug. The calculated EE values for L-ICG-Lipo and S-ICG-Lipo were 65.7 ± 1.2% and 66.9 ± 1.5%, respectively. Both liposomes were concentrated to an ICG concentration of ~100 μg/mL using 30,000 MWCO centrifuge filters (Millipore, Burlington, MA, USA) in a table centrifuge (LC-LX-H185C, LICHEN, Shanghai, China) operating at 4000× *g*.

### 2.4. Characterization of Liposomes

A UV–vis spectrophotometer was utilized to measure the absorbance curves of free ICG and ICG-loaded liposome solutions. The diameter, PDI, and zeta potential of L-ICG-Lipo and S-ICG-Lipo were determined using DLS with a Zetasizer instrument (Nano ZS ZEN3600, Malvern, UK) after a 1:20 dilution with ultrapure water. Three independent measurements were performed to obtain the average values of parameters. The morphologies of L-ICG-Lipo and S-ICG-Lipo were visualized using TEM (Talos™F200X S/TEM, Thermo Fisher Scientific, Waltham, MA, USA). For TEM analysis, a 20 μL drop of the liposome solution was placed on a 200-mesh copper grid, followed by a 20 μL drop of a 2% phosphotungstic acid negative staining solution. After 30 min, the excess staining solution was removed using a 30 μL drop of ultrapure water. The sample was then air-dried at room temperature for a minimum of 12 h prior to TEM measurement.

### 2.5. Animal Models

All animal experiments conducted in this study (2022020641) received approval from the animal ethics committee of Central South University, China. Female BALB/c mice, aged six weeks, were acquired from Hunan SJA Laboratory Animal Co., Ltd., Changsha, China, and were utilized to establish a tumor model using 4T1 cells (mouse breast cancer cells). For the induction of the 4T1 tumor model, each BALB/c mouse received a subcutaneous injection of two million 4T1 cells in the mammary fat pads.

### 2.6. In Vitro and In Vivo PACT

In our previous work, we reported a ring-array-based PACT system [32]. Based on this, we further enhanced the laser coupling efficiency and improved the imaging algorithms, resulting in our new system with faster imaging speed and higher imaging resolution. For example, laser collimation with plano-convex and plano-concave cylindrical mirrors was applied to improve the coupling transmission efficiency of the laser to the fiber. For the imaging algorithm, we used a GPU-accelerated algorithm to realize fast image reconstruction. Our designed PACT system is composed of a Verasonics ultrasound study platform (Vantage 256, Verasonics, Kirkland, WA, USA), a digital delay/pulse generator (DG645, Stanford Research Systems, Sunnyvale, CA, USA), an optical parametric oscillator (OPO) laser (SpitLight 600, InnoLas Laser GmbH, Krailling, Germany), a 5 MHz ring-array transducer (ULSO TECH Co., Ltd., Xingtai, China), and a step motor (Zolix, Beijing, China). To achieve a self-focusing anxiety of 50 mm, each element of the transducer was machined into an arc (with a radius of 50 mm) in the elevation direction. The pitch between the elements was 0.618 mm and inter-element spacing was 0.055 mm. To examine the PA signal consistency of concentrated L-ICG-Lipo and S-ICG-Lipo (containing ~100 μg/mL ICG), in vitro PACT was employed on L-ICG-Lipo and S-ICG-lipo embedded in plastic tubes. To evaluate the penetration ability of the prepared L-ICG-Lipo and S-ICG-Lipo, in vivo PACT was conducted to directly monitor the nanocarrier distribution in tumor sites. In order to validate the feasibility of the PACT system, we only used one mouse per group for imaging experiments. Animals were under continuous anesthesia throughout the PA imaging experiment. The mice were anesthetized with 1% isoflurane using a small-animal anesthesia machine (RWD, R500). In order to optimize the quality of PA imaging, hair removal cream was carefully applied to remove partial hair of the 4T1 tumor-bearing mice. Following this, 200 μL of concentrated L-ICG-Lipo or S-ICG-Lipo (containing ~100 μg/mL ICG) was intravenously injected into the tail vein of the 4T1 tumor-bearing mice. For comparative analysis, the mice were immobilized in a consistent position within a water tank, and PA images were captured before and after the administration of liposomes. L-ICG-Lipo and S-ICG-Lipo were concentrated using 30,000 MWCO centrifuge filters in a table centrifuge operating at 4000× *g*. The PA detections with 780 nm and 1064 nm laser wavelengths were performed to image the ICG-loaded liposomes and the mouse tissues, respectively. The cross-sectional PA images of tumors were obtained at 5 min, 15 min, 30 min, 60 min, 120 min, and 240 min after liposome administration. The PA signal was obtained at the sampling rate of 62.5 MHz and reconstructed using the delay and sum algorithm. All PA images were reconstructed in the Verasonics system and post-processed by MATLAB R2020a (MathWorks, Natick, MA, USA).

### 2.7. Statistical Analysis

Data analysis was conducted using Origin 2021 software. The values presented in the graphs represent means ± standard deviation (s.d.). The comparisons of diameter, PDI, zeta potential, and PA amplitude were performed using Student’s *t*-test. Statistical significance was considered for *p*-values below 0.05 (* *p* < 0.05, ** *p* < 0.01, and *** *p* < 0.001).

## 3. Results and Discussion

### 3.1. Fabrication of Microfluidic Mixing Chip

**Figure 1:**
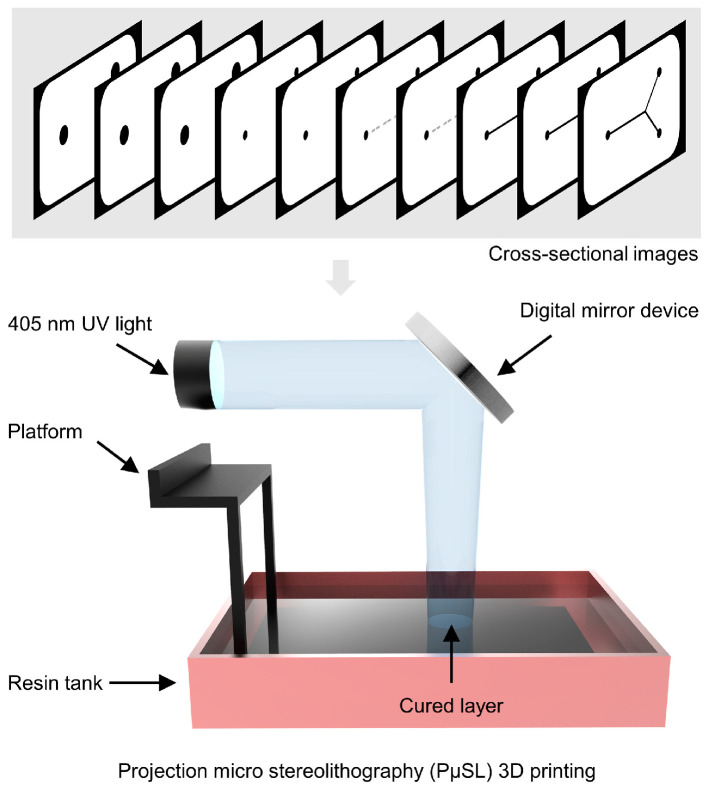
Schematic illustration of the PμSL 3D printing process for fabricating a microfluidic mixer.

3D printing is an emerging additive manufacturing technology that offers numerous advantages, including high production efficiency, flexible design capabilities, and cost-effectiveness [33]. In this study, a high-resolution projection micro stereolithography (PμSL) 3D printer was utilized to create the microfluidic mixer, as depicted in Figure 1 and Figure S2. PμSL represents a cutting-edge 3D printing technique that operates through photopolymerization processes, wherein a photocurable resin is solidified in a layer-by-layer fashion to form the desired device (cross-sectional images in Figure S3). To fabricate high-resolution structures, the thickness of the printing layer was set as 20 μm. In addition, we selected a transparent and rigid resin for fabrication, which was convenient for observations. In contrast to traditional approaches, PμSL offers a significantly simplified fabrication process by circumventing intricate photolithography, casting, and bonding procedures. However, the utilization of 3D-printed microfluidic chips still suffers from challenges, including issues such as over-curing, clogging, and cleaning, particularly as channel size decreases and channel complexity increases. In order to address these concerns, we have fabricated a 3D-printed microfluidic mixing device with open channels, which facilitates easy fabrication and cleaning processes. After cleaning, we used a microfluidic tape as a substrate to seal the open microchannels.

### 3.2. Preparation of L-ICG-Lipo and S-ICG-Lipo

**Figure 2:**
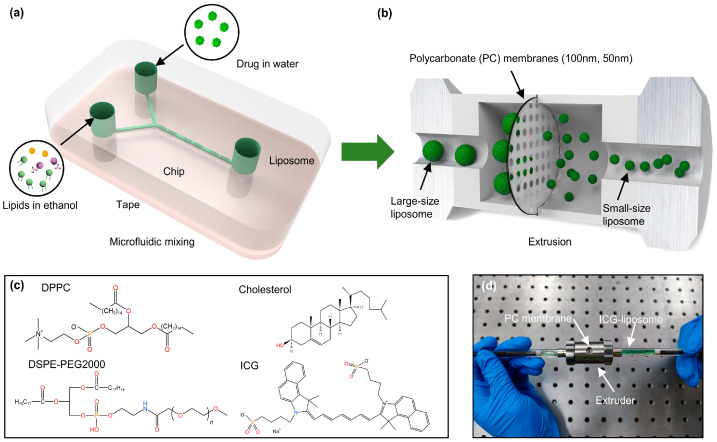
Preparation of L-ICG-Lipo and S-ICG-Lipo. (**a**) Schematic illustration of the formation of L-ICG-Lipo using microfluidic mixing. (**b**) Schematic illustration of the generation of S-ICG-Lipo by extrusion. (**c**) Chemical structures of ICG-loaded liposome components. (**d**) Photograph of the extrusion process.

First, we used the microfluidic mixer to synthesize L-ICG-Lipo (Figure 2a). Ethanol-containing lipids and PBS-containing ICG were mixed in the microchannel with a buffer:ethanol FRR of 4 and a TFR of 10 mL/h. Due to the use of microfluidic tape, we used a low TFR to avoid the leakage of the solution. The microchannel within this device possesses a width of 200 μm and a depth of 150 μm (detailed parameters provided in Figure S4). Through the implementation of the stagger herringbone mixer, the convection–diffusion of the organic phase and buffer is enabled, leading to localized dilution of the organic phase, and subsequently facilitating the rapid formation of self-assembled liposomes. To generate S-ICG-Lipo, we could have reduced size by increasing the FRR. However, a high aqueous-to-organic FRR is known to reduce liposome concentration, which hinders their applications (Shepherd et al., 2021 [23]). We explored an alternative scenario to produce S-ICG-Lipo. We first rapidly prepared the L-ICG-Lipo by using the microfluidic mixer. Next, the S-ICG-Lipo was prepared by using a liposome extruder with 0.1 and 0.05 μm PC membrane filters (Figure 2b). Figure 2c shows the constituents of ICG-loaded liposomes including DPPC, DSPE-PEG2000, and cholesterol. All the components have been employed in FDA-approved products or clinical trials (Wood et al., 2021 [28]). After extrusion, S-ICG-Lipo became clearer compared with L-ICG-Lipo, indicating a significant reduction in particle size (Figure 2d).

### 3.3. Properties of L-ICG-Lipo and S-ICG-Lipo

**Figure 3:**
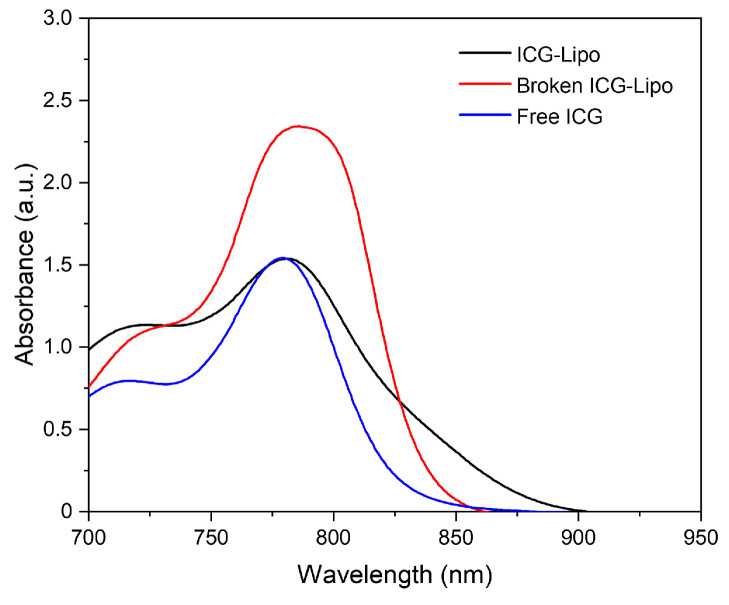
UV–vis spectra of free ICG, ICG-loaded liposome, and broken ICG-loaded liposome.

**Figure 4:**
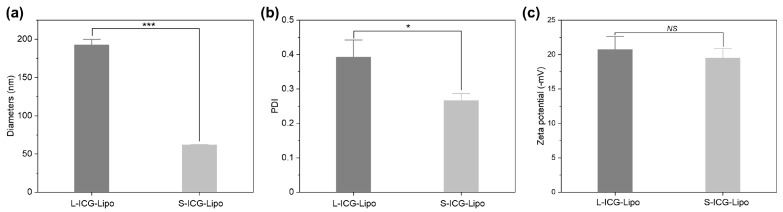
Comparisons of (**a**) diameter, (**b**) PDI, and (**c**) zeta potential in L-ICG-Lipo and S-ICG-Lipo groups. Statistical significance was considered for *p*-values below 0.05 (* *p* < 0.05 and *** *p* < 0.001).

**Figure 5:**
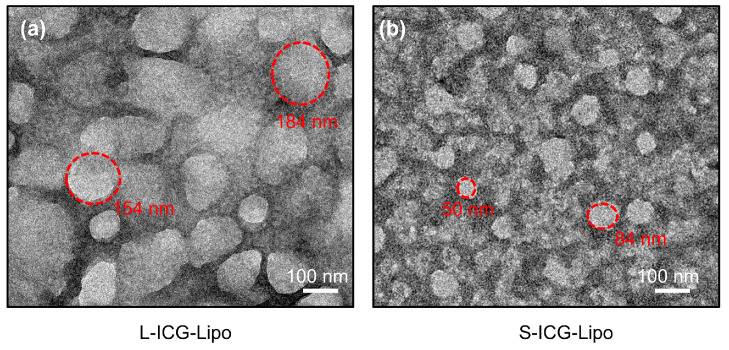
TEM images of (**a**) L-ICG-Lipo and (**b**) S-ICG-Lipo.

We first measured the absorbance curves of free ICG and ICG-loaded liposome solutions (Figure 3) using a UV–vis spectrophotometer. The ICG-loaded liposomes were broken by diluting the purified products with ethanol. With the leakage of ICG from liposomes, the absorbance at 770–830 nm of broken ICG-loaded liposomes was significantly increased, suggesting the successful preparation of liposomes. We further compared the size distribution of L-ICG-Lipo and S-ICG-Lipo. Dynamic light scattering (DLS) results demonstrated that L-ICG-Lipo and S-ICG-Lipo had a mean diameter of 192.6 ± 8.0 and 61.9 ± 0.6 nm, respectively (Figure 4a). Compared with L-ICG-Lipo, S-ICG-Lipo had a smaller polydispersity index (PDI) value, indicating a narrower size distribution (Figure 4b). The zeta potential values of L-ICG-Lipo and S-ICG-Lipo were −20.7 ± 1.9 and −19.5 ± 1.4 mV, respectively, and no difference was observed between zeta potential (Figure 4c). To further validate the formation of S-ICG-Lipo, the morphologies of L-ICG-Lipo and S-ICG-Lipo were observed by transmission electron microscopy (TEM), demonstrating a pronounced difference in liposome size (Figure 5).

### 3.4. The Design of PACT System

**Figure 6:**
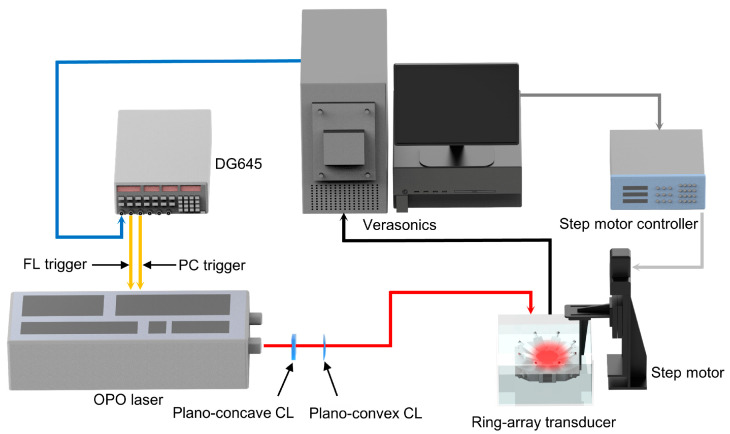
Schematic of the designed PACT system. During illumination, all lasers fire at 20 Hz. Verasonics: ultrasound study platform; DG645: digital delay/pulse generator; OPO laser (10 ns pulse duration, 20 Hz); CL: cylindrical lenses.

As shown in Figure 6, our designed PACT system consists of a Verasonics ultrasound study platform, a digital delay/pulse generator, an optical parametric oscillator (OPO) laser, a 512-element full-ring ultrasonic transducer array with a lateral resolution of 0.1 mm (detailed parameters provided in Figure S5), and a step motor. To achieve a more uniform light distribution of the laser beam, we used eight circumferential distributed fibers to transmit the laser. Besides, we adopted 1064 nm and 780 nm wavelengths to obtain the tissue signal and drug signal, respectively. The 1064 nm wavelength is commonly chosen for PACT due to its deeper tissue penetration capabilities. Longer wavelengths experience less scattering and absorption in biological tissues, allowing for better imaging of structures located at greater depths. The 780 nm wavelength is selected to take advantage of the optical absorption characteristics of ICG. The dual-wavelength PA imaging approach can efficiently unmix background tissue signal and signal from liposomes, offering higher sensitivity and selectivity in tumor sites. Although PACT has demonstrated potential in diverse preclinical contexts, its efficacy in imaging in vivo organs is constrained by several key limitations, namely depth penetration, trade-offs in spatial resolution, and restricted anatomical contrast. Consequently, we believe that investigating these limitations represents a crucial avenue for future research in the examination of pharmacokinetic behavior through PACT methodologies.

### 3.5. Evaluating the Penetration Ability of L-ICG-Lipo and S-ICG-Lipo

**Figure 7:**
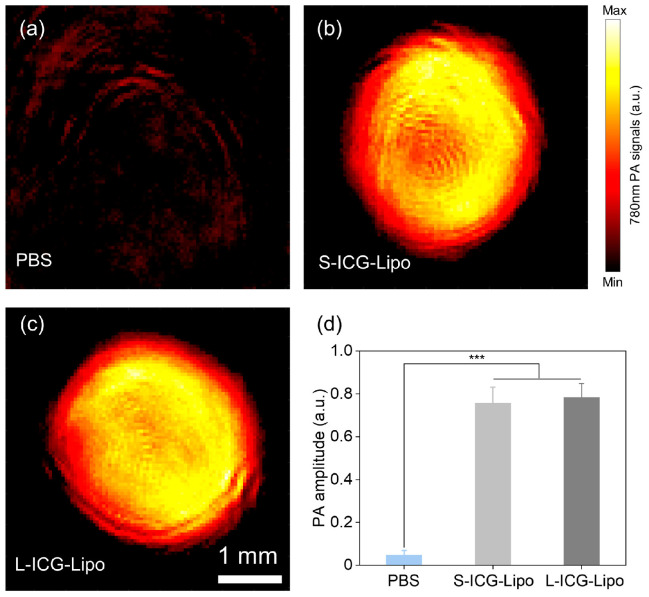
The evaluation of PA performances of L-ICG-Lipo and S-ICG-Lipo. (**a**–**c**) PA images of PBS, S-ICG-Lipo, and L-ICG-Lipo. (**d**) Comparison of PA amplitudes of PBS, S-ICG-Lipo, and L-ICG-Lipo. Statistical significance was considered for *p*-values below 0.05 (*** *p* < 0.001).

**Figure 8:**
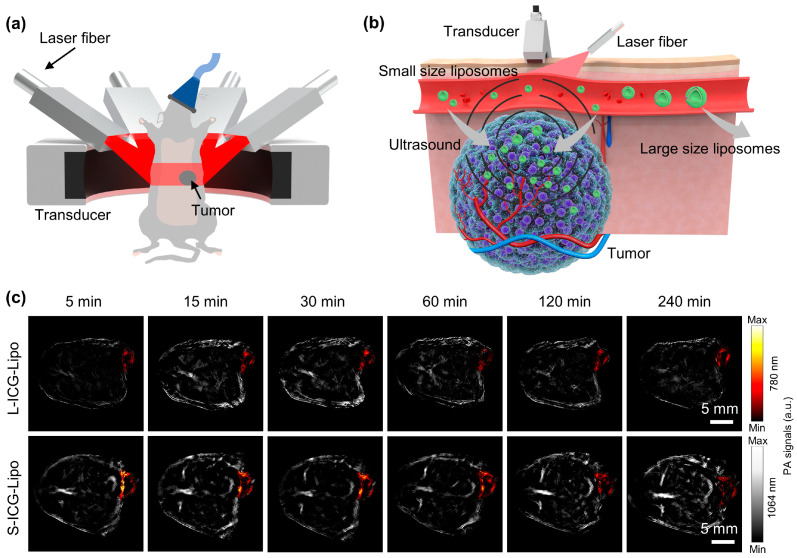
In vivo PACT study. (**a**) PACT workflow: Mice were placed in the center of the ring-array and circumferential distributed fibers emitted lasers on the tumor site. Then, PA signals were detected by ring array, and PA images were reconstructed. Mice were maintained under anesthesia from the time of liposome injection until the completion of imaging. (**b**) Schematic of PACT detection of L-ICG-Lipo and S-ICG-Lipo in tumor sites. (**c**) Representative cross-sectional PA images obtained at 5 min, 15 min, 30 min, 60 min, 120 min, and 240 min points in L-ICG-Lipo and S-ICG-Lipo groups.

**Figure 9:**
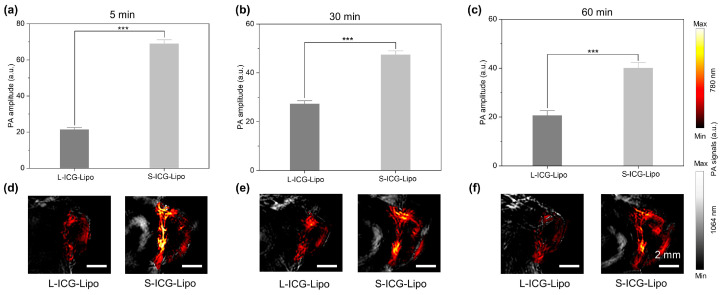
Quantitative analysis of the PA amplitude in tumor sites. (**a**–**c**) The comparisons of PA amplitude of L-ICG-Lipo and S-ICG-Lipo in tumor sites at 5 min, 30 min, and 60 min points. (**d**–**f**) Representative PA images of the tumor region obtained at 5 min, 30 min, and 60 min points in L-ICG-Lipo and S-ICG-Lipo groups. Statistical significance was considered for *p*-values below 0.05 (*** *p* < 0.001).

In this work, we employed a strategy by combining microfluidic mixing with extrusion, achieving the rapid formation of L-ICG-Lipo and S-ICG-Lipo. We first assessed the PA imaging capabilities of L-ICG-Lipo and S-ICG-Lipo in vitro. As shown in Figure 7a–c, PBS, S-ICG-Lipo, and L-ICG-Lipo were placed into three plastic tubes and then imaged using a 780 nm laser. The results suggested that there was no significant difference between S-ICG-Lipo and L-ICG-Lipo (Figure 7d). Compared with PBS, both L-ICG-Lipo and S-ICG-Lipo showed strong PA signals. Then, we evaluated the in vivo penetration ability of L-ICG-Lipo and S-ICG-Lipo by using the PACT system. S-ICG-Lipo and L-ICG-Lipo were administered intravenously to 4T1 (mouse breast cancer) tumor-bearing mice with tumors measuring approximately 5 mm in diameter. The mice were then imaged using the ring-array-based PACT system (Figure 8a). To monitor the tumor size in real time, a magnetic base was used to keep mice in a stationary position. Figure 8b is a schematic of PACT imaging L-ICG-Lipo and S-ICG-Lipo in tumor sites. The PA images from the L-ICG-Lipo and S-ICG-Lipo groups are presented in Figure 8c. The cross-sectional images of tumors were acquired at specific time intervals (5 min, 15 min, 30 min, 60 min, 120 min, and 240 min) after the administration of liposomes. All PA images were reconstructed using a Verasonics system and standardized color bars. The results demonstrated that both L-ICG-Lipo and S-ICG-Lipo exhibited rapid accumulation within the tumor tissues. Compared to L-ICG-Lipo, S-ICG-Lipo showed a more pronounced accumulation behavior in tumor tissues. For quantitative analysis, we quantified the PA amplitudes in the region of interest (ROI) of the tumor sites (Figure 9). All statistical data come from 5 times different acquisitions. At 5 min, 30 min, and 60 time points, we found that S-ICG-Lipo had a higher drug delivery efficiency, suggesting an enhanced tumor penetration ability. Notably, S-ICG-Lipo could rapidly accumulate in the tumor tissues and showed a longer retention performance. The results indicate that our method can efficiently and rapidly evaluate the tumor penetration performance of liposomal nanocarriers with different sizes. Unlike therapeutic studies where multiple mice per group were used, we used only one mouse per group in the imaging experiment. However, it is important to acknowledge that the obtained PA imaging results are relatively preliminary due to the inability of the system to ensure consistent conditions for simultaneous imaging of multiple mice.

## 4. Conclusions

In this study, we developed an ICG-loaded liposomes-assisted PCAT system for monitoring the in vivo penetration ability of drug-loaded liposomes with varying sizes. By combining microfluidic mixing with extrusion, we successfully produced L-ICG-Lipo (192.6 ± 8.0 nm) and S-ICG-Lipo (61.9 ± 0.6 nm). The properties of L-ICG-Lipo and S-ICG-Lipo were estimated by using UV–vis, DLS, and TEM. To validate the practicability of our method, we conducted an in vivo PACT experiment to directly evaluate the penetration ability of L-ICG-Lipo and S-ICG-Lipo in tumor tissues. The results demonstrated that S-ICG-Lipo with a sub-70 nm size had an enhanced penetration performance in tumor sites. Our study offers a dependable approach to directly assess the in vivo penetration capability of liposomal nanocarriers, which has the potential to advance the development of enhanced drug delivery systems.

## Data Availability

The data presented in this study are available on request from the corresponding author. The data are not publicly available due to privacy.

## Supplementary Materials

The following supporting information can be downloaded at: https://www.mdpi.com/article/10.3390/mi15010090/s1, Figure S1: Calibration curve obtained by measuring the absorbance values at 780 nm of samples containing increasing ICG concentrations; Figure S2: The photograph of the PμSL 3D printer; Figure S3: Cross-sectional images of the 3D model; Figure S4: Detailed parameters of the microfluidic mixer; Figure S5: Detailed parameters of the ring-array transducer.
