# Supplementary material for: Indocyanine Green-Loaded Liposomes-Assisted Photoacoustic Computed Tomography for Evaluating In Vivo Tumor Penetration of Liposomal Nanocarriers
Source: Micromachines (Basel). 2023 Dec 30;15(1):90. doi: 10.3390/mi15010090 (PMC10820658; doi:10.3390/mi15010090)
Supplement: Supplementary file 1 [file micromachines-15-00090-s001.zip › micromachines-2656662-supplementary.pdf]

Supplementary Materials

Indocyanine Green-Loaded Liposomes-Assisted Photoacoustic Computed Tomography for Evaluating In Vivo Penetration Ability of Liposomal Nanocarriers

Chenjun Wu,[1,2] Qi Sun,[1] Xiangdong Liu,[1] Xin Sun,[3] Zeyu Chen,[1] and Han Shan*[1]*

[1]State Key Laboratory of Precision Manufacturing for Extreme Service Performance, College of Mechanical and Electrical Engineering, Central South University, Changsha 410083, China

[2]Changjun Riverside Middle School, Changsha 410023, China

[3]Department of Biomedical Engineering, University of Southern California, Los Angeles, CA 90089, USA

*Corresponding authors: Han Shan (hanshancsu@126.com)

Xin Sun (xsun7861@usc.edu)

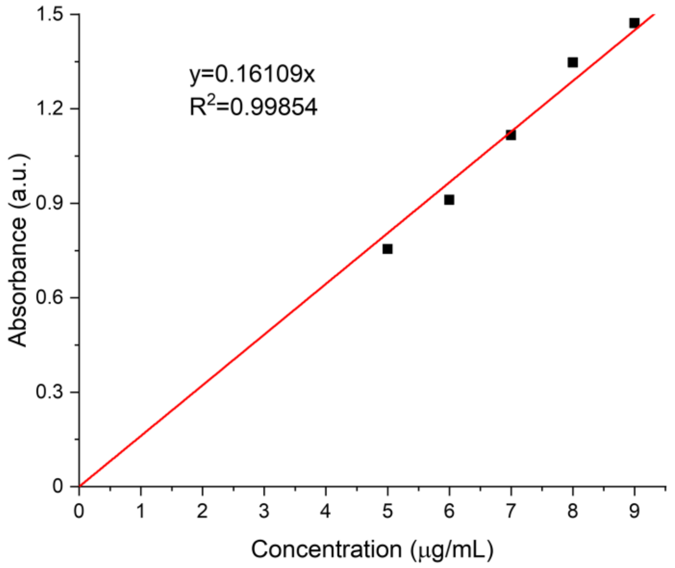

Figure S1. Calibration curve obtained by measuring the absorbance values at 780 nm of samples containing increasing ICG concentrations.

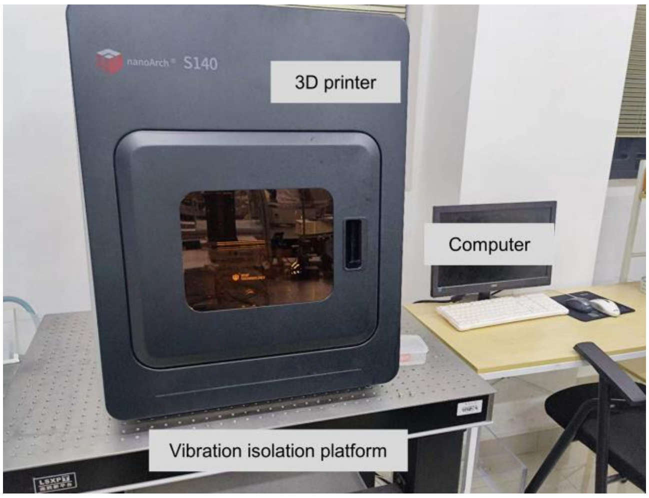

Figure S2. The photograph of the PμSL 3D printer.

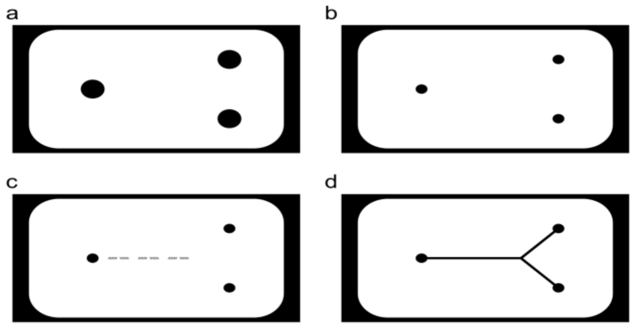

Figure S3. Cross-sectional images of the 3D model.

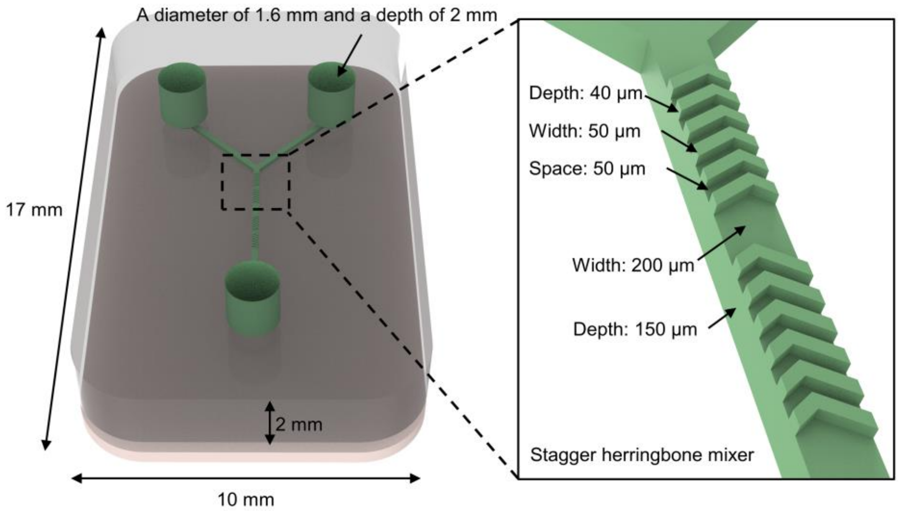

Figure S4. Detailed parameters of the microfluidic mixer.

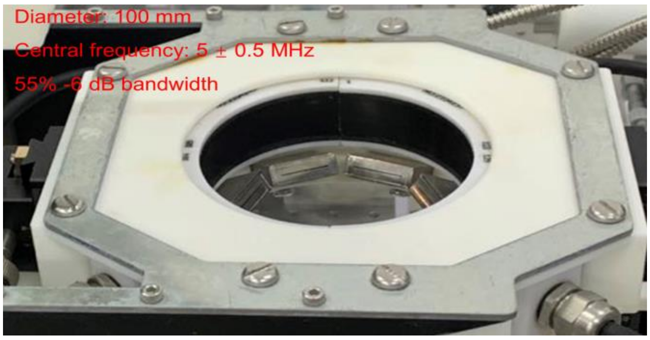

Figure S5. Detailed parameters of the ring-array transducer.
